# Incidental Appendiceal Neuroendocrine Tumor Post Appendectomy: Surgery Is Here to Stay

**DOI:** 10.7759/cureus.78700

**Published:** 2025-02-07

**Authors:** Jerry Kourkoumelis, Haitham Siag, Malia Loustalot, Shani K Palmer

**Affiliations:** 1 Surgery, St. George's University School of Medicine, New York, USA; 2 Surgery, Montefiore New Rochelle Hospital, New Rochelle, USA

**Keywords:** appendectomy, appendicitis, colorectal surgery, general surgery, neuroendocrine tumor, surgical oncology

## Abstract

Neuroendocrine tumors (NETs) arising from the appendix are rare neoplasms but carry significant consequences if missed. These malignancies are typically diagnosed after an appendectomy via histopathological evaluation of the appendix. This aspect further solidifies surgery's place in the treatment of appendicitis.

A 25-year-old female patient presented to the emergency department with a three-day history of right-sided abdominal pain associated with nausea and two episodes of non-bilious vomiting. Physical examination was initially benign but later showed tenderness to the right of the umbilicus. A CT scan revealed an inflamed appendix. Based on clinical and radiological findings, the diagnosis of acute appendicitis was made. The patient underwent a laparoscopic appendectomy. Histopathological analysis of the appendix was performed, identifying an appendiceal neuroendocrine tumor (aNET). Following the initial diagnosis, an appropriate workup was conducted, which included a colonoscopy, computed tomography (CT) scan, and further biopsies.

Histopathological analysis of the appendix revealed a well-differentiated grade 1 NET, measuring 3.5 cm, with invasion into peri-appendiceal tissues. Further evaluation through a repeat CT scan and colonoscopy revealed inflammation in the rectum, cecum, and right colon. Furthermore, a subsequent laparoscopic right hemicolectomy was performed. Pathology of the hemicolectomy specimen revealed no residual NET, though lymph node involvement was present, with three out of 18 nodes testing positive for lymphatic spread.

This case report highlights the diagnostic and management challenges associated with aNETs, emphasizing the importance of surgical intervention in the context of acute appendicitis. The discovery of aNETs can significantly alter the clinical management course, as it did for this patient, who required further surgical intervention and ongoing surveillance. The timely identification and removal of the tumor likely improved the patient’s prognosis.

## Introduction

Neuroendocrine tumors (NETs) arising from the appendix are rare neoplasms. A study conducted from 2014 to 2019 looking at 360 hospitals across the United States of America showed an overall prevalence of seven in 100,000 people [1]. While appendicitis is a common surgical emergency, with a risk of approximately 8.6% in men and 6.7% in women in their lifetime [2], the occurrence of NETs within the inflamed appendix is an infrequent phenomenon. There is a 1% chance that a specimen from an appendectomy is identified as cancerous, with an even smaller percentage being identified as appendiceal neuroendocrine tumors (aNETs) [3]; aNETs are most commonly detected in patients aged 50-80 years old [1]. Despite the rarity of these tumors, their identification is of paramount importance due to the potential for metastasis and the varied clinical presentations they may exhibit.

Understanding the prevalence and incidence of appendicitis provides context for the emergence of NETs. Appendicitis remains one of the most prevalent surgical conditions worldwide, with an annual incidence of approximately 229.9 cases per 100,000 individuals in 2019 [3]. Although appendicitis is typically managed surgically, medical management with antibiotics has been on the rise, especially during the COVID-19 pandemic. There have been recent studies stating that medical management of “uncomplicated” appendicitis can be just as effective as surgical management [4,5]. 

Surgical management of appendicitis should remain the principal treatment in certain clinical settings. Laparoscopic removal of the inflamed appendix not only resolves the acute inflammatory process but also mitigates the risk of potential complications such as perforation and abscess formation [6]. Additionally, a laparoscopic appendectomy is a relatively low-risk procedure involving the removal of a relatively nonessential part of the human body. Moreover, an appendectomy eliminates the need for a later surgical intervention in the case of recurrent appendicitis, as it is 99.4% effective [6]. Importantly, surgical resection facilitates the histopathological examination of the specimen, enabling the detection of incidental findings such as neuroendocrine tumors and other cancers.

This case report highlights the diagnostic and management challenges associated with aNETs, emphasizing the importance of surgical intervention in the context of acute appendicitis.

## Case presentation

A 25-year-old female patient with no past medical history or past surgical history presented to the ED complaining of a three-day history of right-sided flank and abdominal pain, described as a burning sensation with the pain rated an eight on 10. This is the first occurrence of this pain and is associated with nausea and two episodes of non-bilious vomiting.

**Figure 1:**
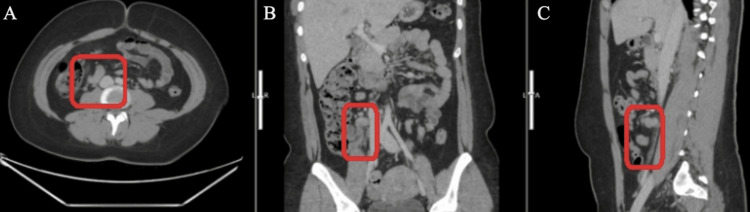
Computed tomography scan of the appendix in the axial (A), coronal (B), and sagittal (C) views The appendix appears thickened and inflamed, dilated up to 16 mm with prominent nearby lymph nodes.

All labs and vitals were within normal limits. The white blood cell count was 8.1 x 10^3. Computed tomography (CT) imaging with IV contrast (100 mL of Omnipaque) of the abdomen and pelvis was done. The CT depicted a non-obstructing punctate left renal stone (on the opposite side of the complaint) as well as a thickened and inflamed appendix measuring up to 16 mm. Also, there were prominent lymph nodes adjacent to the appendix (Figure 1).

Abdominal exam upon first surgical assessment showed a soft, non-tender abdomen with no distension, no rigidity, and no guarding, (-) Rovsing, Psoas sign, and (-) McBurney's, and no tenderness to palpation in the right lower quadrant (RLQ).

Approximately three hours later, upon the assessment of the supervising attending tenderness to deep palpation to the right of the umbilicus was noted.

Using the diagnostic criteria for CT as well as correlating clinically, the diagnosis of acute appendicitis was made. Surgical management was recommended, and the decision was to pursue a laparoscopic appendectomy.

**Figure 2:**
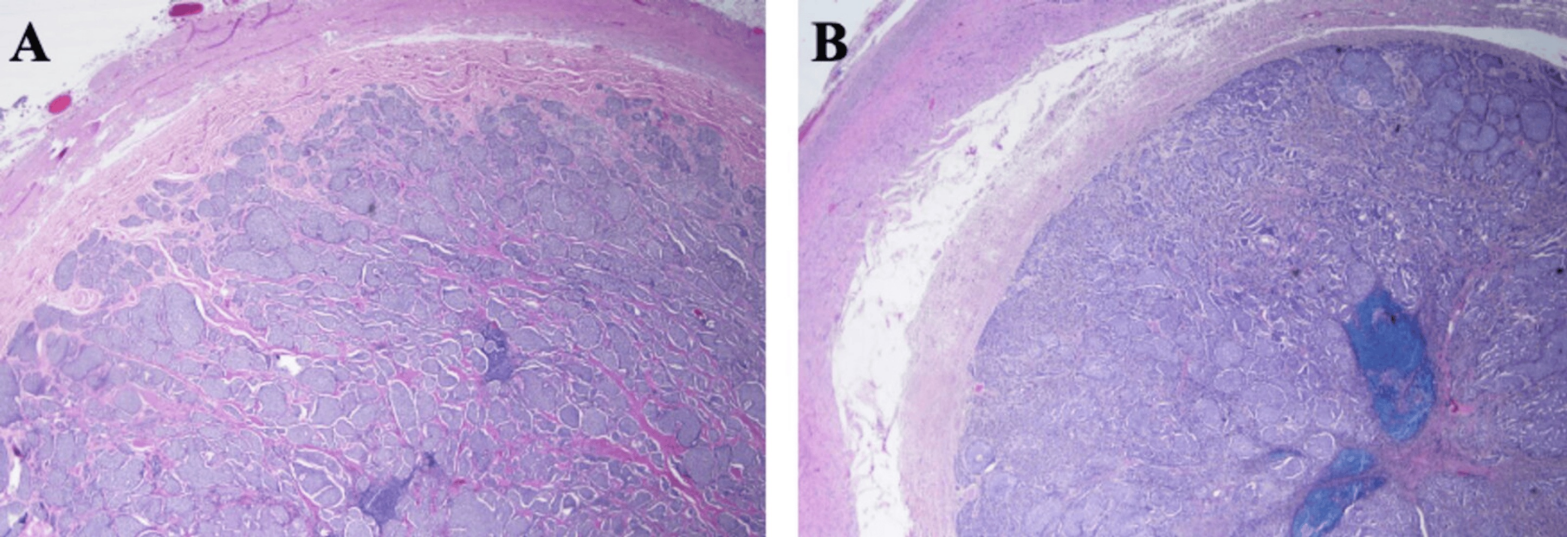
Labels A and B display the entire cross-section of the appendix, showing the lumen filled with nests of the neuroendocrine tumor. Label A, the 20X magnification, shows residual lymphoid aggregates in the center, while label B is the 100X magnification.

**Figure 3:**
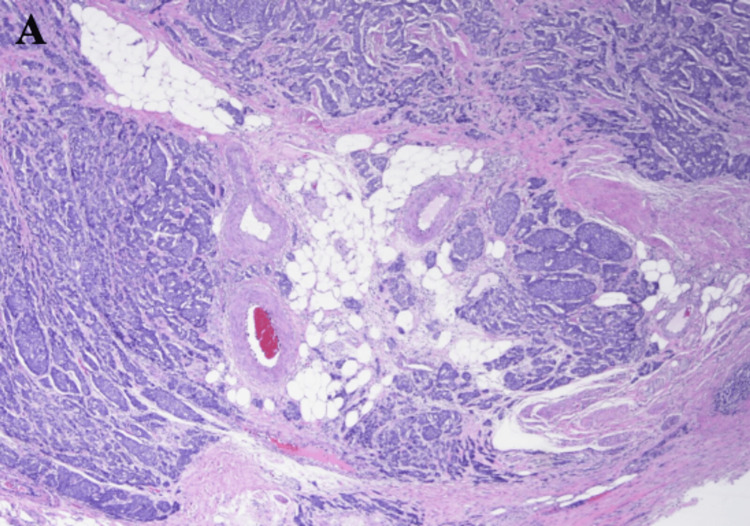
Label A depicts the neuroendocrine tumor infiltrating into the periappendiceal adipose tissue at 20x magnification.

**Figure 4:**
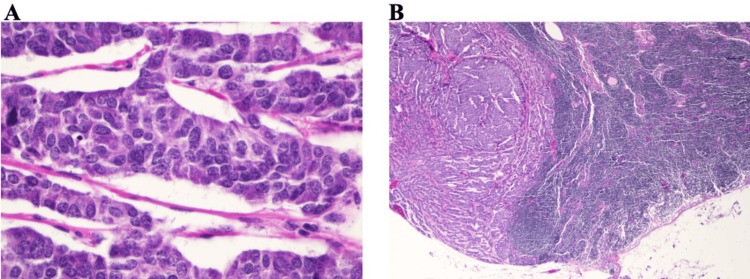
Labels A and B depict a lymph node partially replaced by a tumor. The nests' neuroendocrine tumor cells appear more eosinophilic on the left side of the micrograph. Sheets of lymphocytes appear more blue on the right of the photograph. Label A is the 600x magnification, and label B is the 40x magnification of said invasive cells.

The following pathology report indicated a G1, well-differentiated NET, with focal associated necrosis, measuring 3.5 cm at its greatest diameter. Pathology slides from the laparoscopic appendectomy are below (Figures 2-4). There was extensive involvement of the appendiceal wall and focal invasion into peri-appendiceal fibro-adipose tissue. The following treatment was guided by National Comprehensive Cancer Network (NCCN) guidelines [7]. The patient, then, underwent a colonoscopy before her scheduled right hemicolectomy. The colonoscopy revealed inflammation in the rectum, cecum, and right colon.

**Figure 5:**
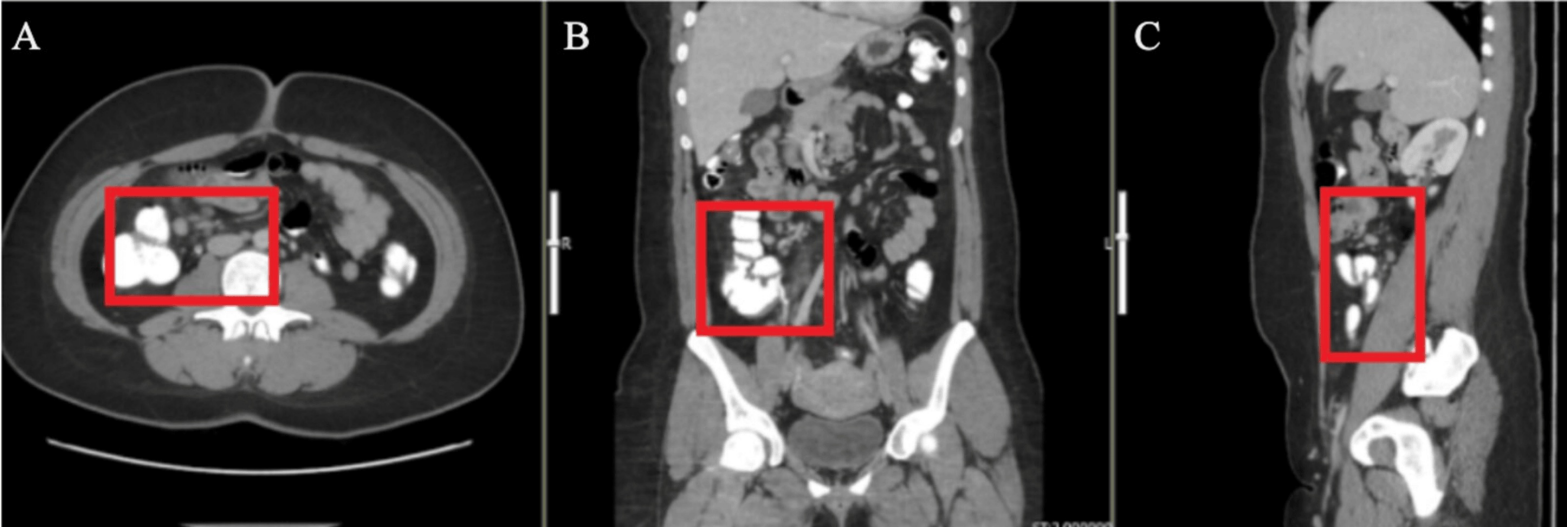
A CT scan showing the axial (A), coronal (B), and sagittal (C) views Status post appendectomy with surgical staple line, mild thickening of the cecal wall, multiple prominent lymph nodes, largest 2.1 cm x 1.5 cm with shotty subcentimeter retroperitoneal lymph nodes.

The patient obtained a repeat CT with IV and oral contrast (Figure 5). She then underwent a laparoscopic right hemicolectomy with ileocolic anastomosis.

The subsequent pathology report revealed a cecum with active colitis and associated mucosal ulceration. There was no residual NET identified in the bowel wall surrounding the site of the previous appendectomy. Proximal and distal resection margins were unremarkable. There were pericolonic soft tissue tumor deposits, large vessel tumor emboli, and perineural tumor invasion all identified in the specimen. Moreover, three out of 18 peri-colonic nodes obtained were positive, depicting a NET with lymph node involvement.

Current management

**Figure 6:**
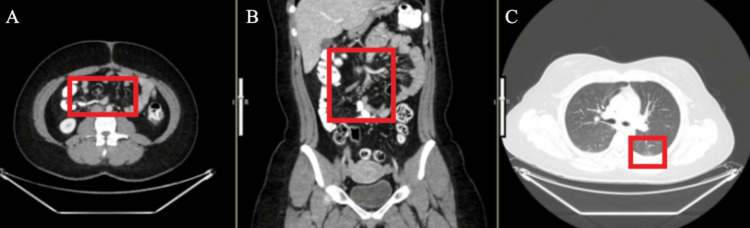
CT scan showing the axial (A), coronal (B) and axial (c) views Scattered tiny retroperitoneal and mesenteric lymph nodes, most are at the root of the mesentery and appear overall somewhat smaller than prior, small vague indeterminate ground glass focus in the left base measuring 11 mm (consider follow-up Fleischner criteria [8]).

The patient is currently undergoing tumor surveillance every six months via CT scans. The most recent CT scans obtained at the time of writing this report are presented in Figure 6.

## Discussion

Medical management of appendicitis typically involves antibiotic therapy and supportive measures, aiming to resolve symptoms without identifying the cause. While this approach may offer relief for certain cases, it inherently carries the risk of recurrence, which in one meta-analysis study was found to be 18.2% [2]. Importantly, medical management may overlook underlying pathologies, such as malignancies, which may present with atypical symptoms of appendicitis.

Conversely, an appendectomy addresses the acute inflammatory process and allows for histopathological examination of the appendix. This aspect becomes crucial in cases like the one presented here, where a stage 3 aNET with three positive lymph nodes was identified. Therefore, surgical intervention offers the advantage of definitive treatment while concurrently enabling the detection and staging of incidental pathologies, thus mitigating the risk of delayed diagnosis and progression to advanced stages.

This is an interesting case due to the lack of associated risk factors such as age, race, and hypertension. This type of tumor is more likely to be found in people aged between 50 and 80 as well as those of the Caucasian population [1], whereas the patient is 25 years old and of Hispanic heritage. Additionally, she does not have any history of primary gastrointestinal malignancy in her family, which is one of the strongest predisposing factors to aNETs, nor does she have hypertension, which was noted to be present in over 40% of the patients [1]. It is important to note that the patient does have the risk factor of being female; however, we believe that this factor alone does not have a large enough impact to classify her as being at risk since it is only a slight preponderance of 54.95% in females as to 45.05% in males [9].

The patient was able to be accurately and promptly diagnosed as having appendicitis. The key decision to manage appendicitis surgically rather than medically might have increased her long-term overall survival. The five-year survival rate for aNETs is approximately 100% for low-stage tumors but falls to 12-28% in the case of distant metastases [10]. If medically managed, the overall inflammation may have gone down, and her mild initial clinical symptoms may have resolved, but her underlying malignancy would not have not. Moreover, the size of the tumor, being 3.5 cm, meant that she also needed a future right hemicolectomy.

## Conclusions

Our patient's case highlights the diagnostic challenges and therapeutic complexities of managing appendiceal malignancies, which can emerge even when the initial presentation appears to be a mild case of acute appendicitis. Despite the rarity of aNETs, her timely diagnosis and surgical intervention emphasize the pivotal role of an appendectomy in both resolving the acute inflammatory process and determining a pathologic diagnosis. At which the patient's prognosis was elucidated, possibly improving her long-term outcome. As we navigate the landscape of appendiceal pathology, surgical management remains a valuable tool in the treatment of acute appendicitis.
